# Antitumor effects of erlotinib in combination with berberine in A431 cells

**DOI:** 10.1186/s40360-023-00661-2

**Published:** 2023-05-11

**Authors:** Xiangdan Cuan, Xingying Yang, Weiwei Zhu, Yue Zhao, Rui Luo, Yanping Huang, Xuanjun Wang, Jun Sheng

**Affiliations:** 1 Key Laboratory of Pu-er Tea Science, Ministry of Education, Yunnan Agricultural University, No. 452, Fengyuan Road, Panlong District, Kunming, 650201 China; 2 College of Science, Yunnan Agricultural University, Kunming, 650201 China; 3 College of Food Science and Technology, Yunnan Agricultural University, Kunming, 650201 China; 4 State Key Laboratory for Conservation and Utilization of Bio-Resources in Yunnan, Kunming, 650201 China; 5 Yunnan Research Institute of Plateau Characteristic Agricultural and Industry, Kunming, 650201 China

**Keywords:** Berberine, Erlotinib, EGFR positive cells, Combination-therapy, A431 cells

## Abstract

**Background:**

First-generation epidermal growth factor receptor tyrosine kinase inhibitors (EGFR-TKIs), such as erlotinib, have been shown to target tumors with L858R (exon 21) and exon 19 deletions, resulting in significant clinical benefits. However, acquired resistance often occurs due to EGFR mutations. Therefore, novel therapeutic strategies for treatment of patients with EGFR-positive tumors are needed. Berberine (BBR) is an active alkaloid extracted from pharmaceutical plants such as *Coptis chinensis*. Berberine has been shown to significantly inhibit EGFR activity and mediate anticancer effects in multiple preclinical studies. We investigated whether combining BBR with erlotinib could augment erlotinib-induced cell growth inhibition of EGFR-positive cells in a mouse xenograft model.

**Methods:**

We examined the antitumor activities and potential mechanisms of erlotinib in combination with berberine *in vitro* and *in vivo* using the MTT assay, immunoblotting, flow cytometry, and tumor xenograft models.

**Results:**

*In vitro* studies with A431 cells showed that synergistic cell growth inhibition by the combination of BBR and erlotinib was associated with significantly greater inhibition of pEGFR and pAKT, and inhibition of cyclin D and Bcl-2 expression compared to that observed in response to BBR or erlotinib alone. The efficacy of the combination treatment was also investigated in nude mice. Consistent with the *in vitro* results, BBR plus erlotinib significantly reduced tumor growth.

**Conclusion:**

Our data supported use of BBR in combination with erlotinib as a novel strategy for treatment of patients with EGFR positive tumors.

## Introduction

Epidermal growth factor receptor (EGFR) tyrosine kinase is a classic receptor tyrosine kinase that mediates cell proliferation in response to multiple ligands [1]. Abnormal activation of EGFR is associated with breast, lung, and colon tumors. Therefore, EGFR is the target of several cancer therapeutics [2, 3]. Epidermal growth factor receptor and related members of the ErbB family, such as ErbB2, ErbB3, and ErbB4, contain a glycosylated extracellular ligand binding domain, and single pass TM domain, and an intracellular juxtamembrane, tyrosine kinase, and autophosphorylation domain [4, 5]. Ligand binding promotes receptor dimerization and activation of intracellular protein tyrosine kinase activity. Epidermal growth factor receptor is the target of several cancer therapeutics including monoclonal antibodies targeted to the extracellular part of EGFR and small molecule inhibitors of the EGFR kinase [6]. Monoclonal antibodies include cetuximab and nimotuzumab. Nimotuzumab (h-R3) is a monoclonal antibody that targets EGFR through binding to the extracellular domain, which inhibits EGF binding [7]. The first generation of EGFR TKIs includes erlotinib, gefitinib, and lapatinib. Erlotinib is a reversible EGFR TKI used to treat NSCLC with in-frame deletions of exon 19 and an exon 21 L858R point activating mutation [8]. Nearly all patients develop resistance to erlotinib within 9–14 months of treatment [9–11]. Non-small cell lung cancer accounts for 85% of all lung cancers and over 60% express WT EGFR [12]. Patients with WT EGFR tumors are relatively insensitive to EGFR TKIs and patients with tumors that express WT EGFR in the absence of other targetable mutations have limited treatment options [13, 14]. The majority of EGFR positive cancers do not respond to TKIs or to mAb. Therefore, development of novel therapeutic strategies is urgently needed. Many recent studies on the efficacy of monotherapies have been severely limited by tumor cell population heterogeneity and redundant growth and survival pathways [15, 16]. Several studies showed that bevacizumab combined with erlotinib prolonged PFS in patients with NSCLC [17, 18]. Zhang et al. showed that EGCG combined with erlotinib synergistically suppressed tumor growth by targeting the common EGFR/AKT signaling pathways [19].

Natural products are widely used and their anti-cancer activities have attracted considerable attention [20, 21]. Berberine (BBR) is an active alkaloid extracted from pharmaceutical plants such as *Coptis chinensis* [22]. Berberine has been extensively studied in chemically induced rodent carcinogenesis models and in several types of cancers including human colon cancer, lung cancer, and breast cancer. Berberine has been shown to inhibit cancer cell growth, induce cell cycle arrest, and promote apoptosis [23–25]. Studies have shown that BBR (from 8.4 to 33.6 µg/mL) reduced the viability of A549 and NCI-H1299 cells in time- and concentration-dependent manners [26]. In addition, BBR was reported to inhibit tumor cell growth through suppression of phosphorylation of EGFR and other signaling mediators such as ERBB2 and VEGF [27]. A previous study showed that BBR combined with irinotecan induced apoptosis in colon cancer cells [28]. In addition, BBR combined with evodiamine synergistically suppressed MCF-7 cell proliferation [29]. These studies indicated that combination therapies may prevent drug resistance and enhance efficacy. Therefore, the goal of this study was to determine whether combination treatment with BBR and erlotinib could exert antitumor effects against A431 cells (EGFR overexpressing, WT).

The results showed that the combination of BBR with erlotinib induced synergistic antitumor effects in A431 cells *in vitro* and *in vivo*, as evidenced by inhibition of cell proliferation and induction of apoptosis.

## Material and methods

### Chemicals and reagents

Erlotinib was purchased from Dalian Meilun Biotechnology Co., Ltd (Dalian, People’s Republic of China). Berberine (> 98% pure) was purchased from MCE (shanghai, People’s Republic of China). Molecular biology grade dimethyl sulfoxide (DMSO) was purchased from VWR Life Science (Atlanta, GA, USA). Dulbecco’s modified Eagle’s medium (DMEM) and all chemicals used for cell culture were purchased from HyClone (Logan, UT, USA). All antibodies were purchased from Cell Signaling Technology (Danvers, MA, USA), except total EGFR, which was purchased from Santa Cruz Animal Health (Santa Cruz, CA, USA). Secondary anti-rabbit and anti-mouse antibodies were purchased from R&D Systems (Minnesota, MN, USA). 3-(4,5-dimethylthiazol-2-yl)-2, 5-diphenyltetrazolium bromide was purchased from Sigma-Aldrich (MTT; St. Louis, MO, USA).

### Cell lines and cell culture

A431 cells were maintained in DMEM culture medium supplemented with 10% fetal bovine serum (FBS; Biological Industries, Ness Ziona, Israel). NCI-H1666, NCI-H441, and NCI-H1781 cells were maintained in 1640 culture medium supplemented with 10% FBS.

### Cell proliferation assay

A431 and NCI-H1975 cells (1.5 × 10^6^ cells/mL) were treated with different concentrations (0, 0.625, 1.25, 2.5, 5, and 10 µg/mL) of BBR and 0.01 µM erlotinib. NCI-H441 cells (1.5 × 10^6^ cells/mL) were treated with different concentrations (0, 2.5, 5, 10, 20, and 40 µg/mL) of BBR and 0.01 µM erlotinib. NCI-H1781 cells (1.5 × 10^6^ cells/mL) were treated with different concentrations (0, 5, 10, 20, 40, and 80 µg/mL) of BBR and 0.01 µM erlotinib. Cells were treated for 48 h. Cell viability was measured spectrophotometrically using the MTT assay in which DMSO was used to dissolve formazan. To evaluate the synergistic effects of BBR and erlotinib, the Q-value was calculated using King’s formula.

### Western blot

A431 cells (2.5 × 10^6^ cells/plate) in 60-mm plates were treated with BBR, erlotinib, or BBR plus erlotinib in a 60 mm plate. After treatment the cells were collected in RIPA lysis buffer containing protease inhibitors (50:1). Cell lysates were centrifuged and the supernatants were collected, mixed with sample loading buffer, and boiled for 10 min. Protein quantification was performed using BCA Protein Assay Reagent according to the manufacturer’s instructions (Beyotime, Shanghai, People’s Republic of China). Western blot analyses were performed following 8–10% SDS-PAGE and transfer to PVDF membranes. After blocking (5% skim milk) for 1 h, membranes were incubated with total EGFR, ERK, AKT, phospho-EGFR, phospho-ERK, and phospho-AKT antibodies, and anti-β-tubulin (1:1,000) at 4 ˚C overnight. The membranes were then treated with secondary anti-rabbit or anti-mouse antibodies (1:5,000) at room temperature for 1 h.

### Colony formation assay

A431 cells (1 × 10^3^ cells/plate) were treated with BBR (2.5 µg/mL), erlotinib (0.01µM), or BBR plus erlotinib in 6-well plates. After 10 days, the colonies were fixed in 4% paraformaldehyde for 15 min and stained with 0.1% crystal violet for 30 min. After washing, images were captured. Crystal violet was dissolved in 2% sodium dodecyl sulfate for 1 h and absorbance was measured at 570 nm.

### Flow cytometry for evaluation of apoptotic cell death

Induction of A431 cell apoptosis by BBR and erlotinib was quantitatively determined by flow cytometry using the Annexin V-conjugated Alexafluor 488 (Alexa488) Apoptosis Detection Kit according to the manufacturer’s instructions (Becton, Dickinson, shanghai, People’s Republic of China). A431 cells were treated with BBR (2.5 µg/mL), erlotinib (0.01µM), or BBR (2.5 µg/mL) plus erlotinib (0.01µM) for 48 h. The cells were harvested, washed with PBS, and incubated with Alexa488 and propidium iodide (PI) for cellular staining at room temperature for 15 min in the dark.

### ***In vivo*** tumor growth

Animal experiments were performed in accordance with ARRIVE guidelines and were approved by the Committee on Animal Handling of Yunnan Agricultural University (YNAU2019LLWYH003-1b). Male athymic nude mice were purchased from Cavens Lab Animal (Changzhou, China). They were housed in the Animal Care Facility in the Key Laboratory of Pu-er Tea Science under a 12 h light-dark cycle (lights on at 9:00 am; lights off at 9:00 pm). Mice had free access to water and food in a room controlled at 22–25 °C and 50–60% humidity. A431 cells (5 × 10^6^) were suspended in 200 µL of normal saline and subcutaneously implanted into 6-7-week-old male athymic nude mice. Tumors began to appear 3 days after the tumor cells were injected. When the tumor volume reached approximately 50 mm^3^, the tumor-bearing mice received daily (five times per week) intraperitoneal injections with vehicle control, BBR (15 mg/kg), erlotinib (25 mg/kg), or the combination of BBR (15 mg/kg) and erlotinib (25 mg/kg) for 26 days. During treatment mouse body weight and tumor volume were recorded every two days. Tumor volume was calculated using the following formula: of A × B^2^ × 0.5 (A is solid tumor length, B is width). After 26 days the mice were euthanized by intraperitoneal injection of sodium pentobarbital. One portion of the tumor was fixed in formalin and the other portion of the tumor was frozen at − 80 °C.

### Immunohistochemical staining (IHC)

Xenograft tumor tissue sections were incubated at 65 °C for 3 h, then deparaffinized and rehydrated. Deparaffinized and rehydrated sections were treated for antigen retrieval using sodium citrate buffer (pH 6.0). The slides were blocked using serum albumin for 20 min at 37 °C. The slides were then incubated with primary antibody at 4 °C overnight. Then, the slides were incubated with secondary antibody for 30 min at 37 °C. Target proteins and cell nuclei were visualized using DAB substrate and hematoxylin, respectively. Slides were visualized using a light microscope. Images were captured using 400× magnification.

### Statistical analysis

All data were analyzed using SPSS17.0 software (mean ± SEM, one-way ANOVA). Student’s t-test was used to determine tumor volume statistical significance. *P* < 0.05 was considered significant, *P* < 0.01 was a significant difference, and *P* < 0.001 was extremely significant. The experimental data were plotted using GraphPad Prism software.

## Results

### Berberine combined with erlotinib decreased proliferation of EGFR positive cells

**Fig. 1:**
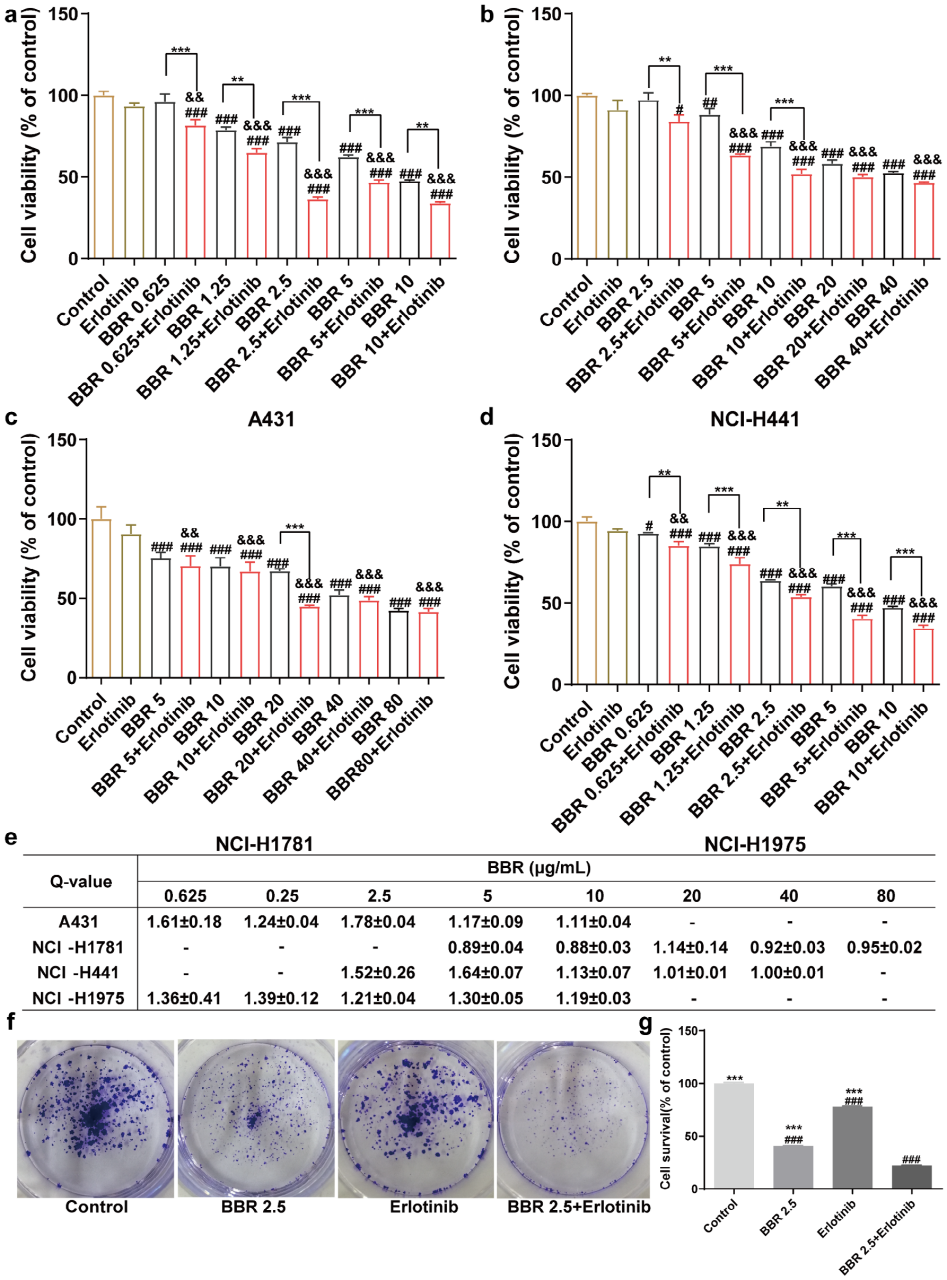
Combination therapy inhibited EGFR positive cell proliferation. (**a**) (**b**) (**c**) (**d**) Endothelial growth factor receptor positive cells were treated with BBR combined with erlotinib for 48 h, and cell viability was determined using the MTT assay. Data are expressed in terms of the percent of control cells (DMSO treated) as the mean ± SEM of 3 replicates. (**e**) Q-value. The Q-value was calculated using King’s formula. (**f**) Colony formation assay was used to determine the inhibitory effect of BBR combined with erlotinib on A431 cells. Representative images of colonies from 60-mm plates in the colony formation assay. (**g**) Quantification of the colony number of A431 cells. Data were analyzed using one-way ANOVA, ^#^*P* < 0.05; ^##^*P* < 0.01; ^###^*P* < 0.001 vs. control. ^*^P < 0.05; ^**^P < 0.01; ^***^P < 0.001 vs. combination-therapy group, ^&^*P* < 0.05; ^&&^*P* < 0.01; ^&&&^*P* < 0.001 vs. erlotinib

To study the sensitivity of EGFR-positive cell lines to BBR and erlotinib, we examined 4 cell lines (A431, NCI-H441, NCI-H1781 and NCI-H1975), of which A431, NCI-H441, and NCI-H1781 cells expressed wild-type EGFR and NCI-H1975 cells had EGFR^L858R/T790M^ double mutation. Epidermal growth factor receptor positive cells were treated with 0 to 80 µg/mL BBR. As shown in Fig. 1a, b and c, and 1d, BBR inhibited growth of all 4 cell lines in a dose-dependent manner at 48 h. A previous report showed that BBR did not induce toxicity in normal human epidermal keratinocytes [28]. Next, we treated with BBR with erlotinib and evaluated cell viability using the MTT assay. As shown in Fig. 1a and d, treatment with BBR and erlotinib was significantly more effective than either agent alone in each of the 4 cell lines. The degree of response to the combination therapy varied between cell lines. Different sensitivities to various treatments were partly attributed to tumor heterogeneity. Berberine and erlotinib decreased cell viability up to 28.69% at 2.5 µg/mL and 7.68% at 0.01µM, respectively (lane 2 and lane 7, Fig. 1a). Moreover, the combination of BBR and erlotinib further decreased cell viability (63.58% inhibition) compared to either agent alone in A431 cells (lane 8, Fig. 1a). Berberine and erlotinib exerted more pronounced synergistic effects in A431 cells than in other cells based on Q-value (Fig. 1e). Berberine and erlotinib monotherapies induced dose-dependent inhibition of A431 cell expansion and 2.5 µg/mL BBR and 0.01µM erlotinib were identified as the best concentrations for assessment of interactions.

These results were further confirmed using a clonogenic assay. Anchorage independent colony formation assays further demonstrated the synergistic effect of BBR and erlotinib on cell proliferation. The results showed that co-treatment with BBR and erlotinib (22.37% survival rate) significantly inhibited NCI-H441 colony formation compared with either BBR (40.79% survival rate) or erlotinib (78.21% survival rate) alone, (Fig. 1f and g). These results indicated that the combination of BBR and erlotinib synergistically inhibited cell growth in A431 cells.

**Fig. 2:**
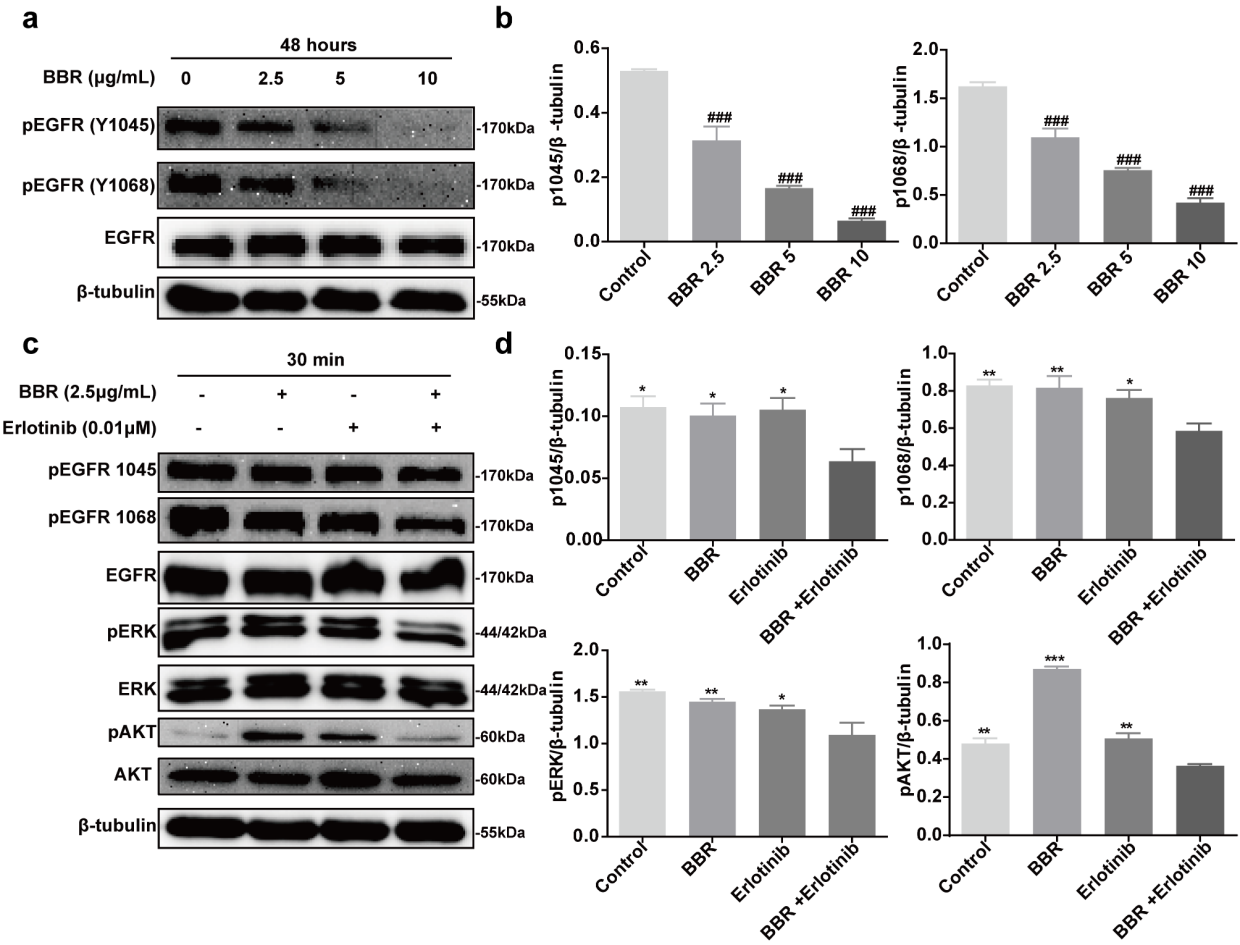
Berberine combined with erlotinib inhibited the EGFR signaling pathway in A431 cells. (**a**) A431 cells were treated with a range of BBR doses for 48 h. Phosphorylation of EGFR, AKT, and ERK were evaluated using western blot. The p-EGFR, p-ERK1/2 p-AKT, EGFR, ERK1/2, AKT, and β-tubulin proteins were separated using 8% SDS-PAGE. Each experiment was performed 3 times independently. (**c**) A431 cells were treated with BBR (2.5 µg/mL) combined with erlotinib (0.01µM) for 30 min. Phosphorylation of each protein was evaluated using western blot. Each experiment was also performed 3 times independently. (**b**, **d**) Quantification of EGFR and downstream signaling proteins. Data were analyzed using one-way ANOVA, ^#^*P* < 0.05; ^##^*P* < 0.01; ^###^*P* < 0.001 vs. control. ^*^P < 0.05; ^**^P < 0.01; ^***^P < 0.001 vs. combination-therapy group

### Berberine combined with erlotinib inhibited the expression of EGFR and its downstream targets

We examined the effects of BBR and erlotinib on EGFR signal transduction in A431 cells using immunoblotting. As shown in Fig. 2a-b, BBR inhibited EGFR phosphorylation in a dose-dependent matter (2.5 to 10 µg/mL) in A431cells. We then evaluated the effects of BBR combined with erlotinib on the EGFR pathway. Western blot analysis showed that 30 min treatment with BBR and erlotinib inhibited the EGFR pathway (*p* < 0.05), as evidenced by reduced phosphorylation of EFGR (Y1045, Y1068), extracellular regulated protein kinases (ERK), and protein kinase B (AKT) (lane 4, Fig. 2c and d). These results indicated that combined treatment with BBR with erlotinib inhibited the EGFR pathway.

### Combined treatment with BBR and erlotinib induced apoptosis in A431cells

**Fig. 3:**
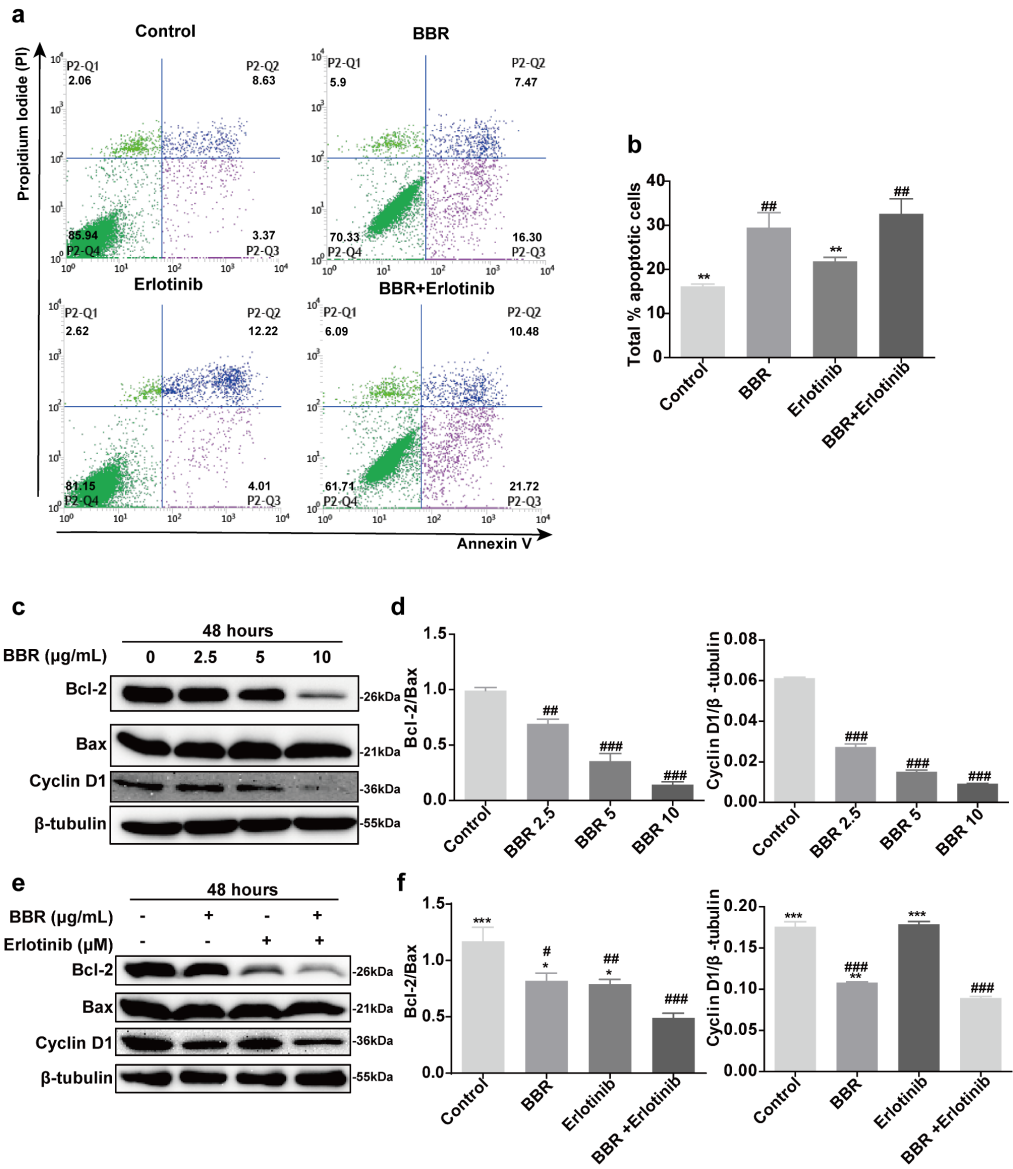
Expression level of apoptosis proteins in response to different drug treatments in A431 cells. (**a**) Berberine (2.5 µg/mL) and erlotinib (0.01 µM) synergistically enhanced apoptosis in A431 cells as determined by flow cytometry using the Annexin V-conjugated Alexafluor 488 (Alexa488) Apoptosis Detection Kit. (**b**) The ratio of apoptotic cells in each group. (**c**) Treatment of A431 cells with varying concentrations of BBR for 48 h. The expression levels of the apoptotic proteins Bcl-2/Bax were determined using western blot. Bcl-2, Bax, and cyclin D1 protein were separated using 10% SDS-PAGE. Each experiment was performed 3 times independently. (**e**) Treatment of A431 cells with BBR (2.5 µg/mL) combined with erlotinib (0.01 µM) for 48 h. The expression of the apoptotic proteins Bcl-2/Bax was determined using western blot. (**d, f**) Quantification of Bcl-2/Bax and cyclin D1 proteins. Each experiment was performed 3 times independently. Data were analyzed using one-way ANOVA, ^#^*P* < 0.05; ^##^*P* < 0.01; ^###^*P* < 0.001 vs. control. ^*^P < 0.05; ^**^P < 0.01; ^***^P < 0.001 vs. combination therapy group

We evaluated the mechanism of combined BBR and erlotinib therapy on A431 cell proliferation and apoptosis. As shown in Fig. 3a-b, A431 cells were treated with BBR, erlotinib, or both for 48 h, then stained with annexin V-FITC/PI and analyzed using flow cytometry. Combination treatment induced more apoptosis (32.63%) than the untreated control (16.15%), BBR (29.47%), or erlotinib (21.84%). We also determined the expression levels of Bax and Bcl-2, which are important in apoptosis. Compared to the untreated control, BBR significantly reduced the Bcl-2/Bax ratio a concentration-dependent manner (Fig. 3c and d). Compared to the untreated control (*p* < 0.001) and the individual drugs (*p* < 0.05), BBR plus erlotinib significantly reduced the Bcl-2/Bax ratio (Fig. 3e and f). These results showed that erlotinib did not effectively induce apoptosis, but the combination of BBR and erlotinib significantly increased apoptotic cell death.

We then evaluated the effects of BBR plus erlotinib on cell cycle progression in A431 cells. Treatment with erlotinib alone had minimal effects on cyclin D in A431 cells (*p* > 0.05). Combined treatment with BBR and erlotinib led to significantly lower cyclin D1 expression than that in cells treated with BBR (*p* < 0.01) or erlotinib (*p* < 0.001, Fig. 3e and f).

### Effect of BBR in combination with erlotinib on tumor growth in A431 xenograft models

**Fig. 4:**
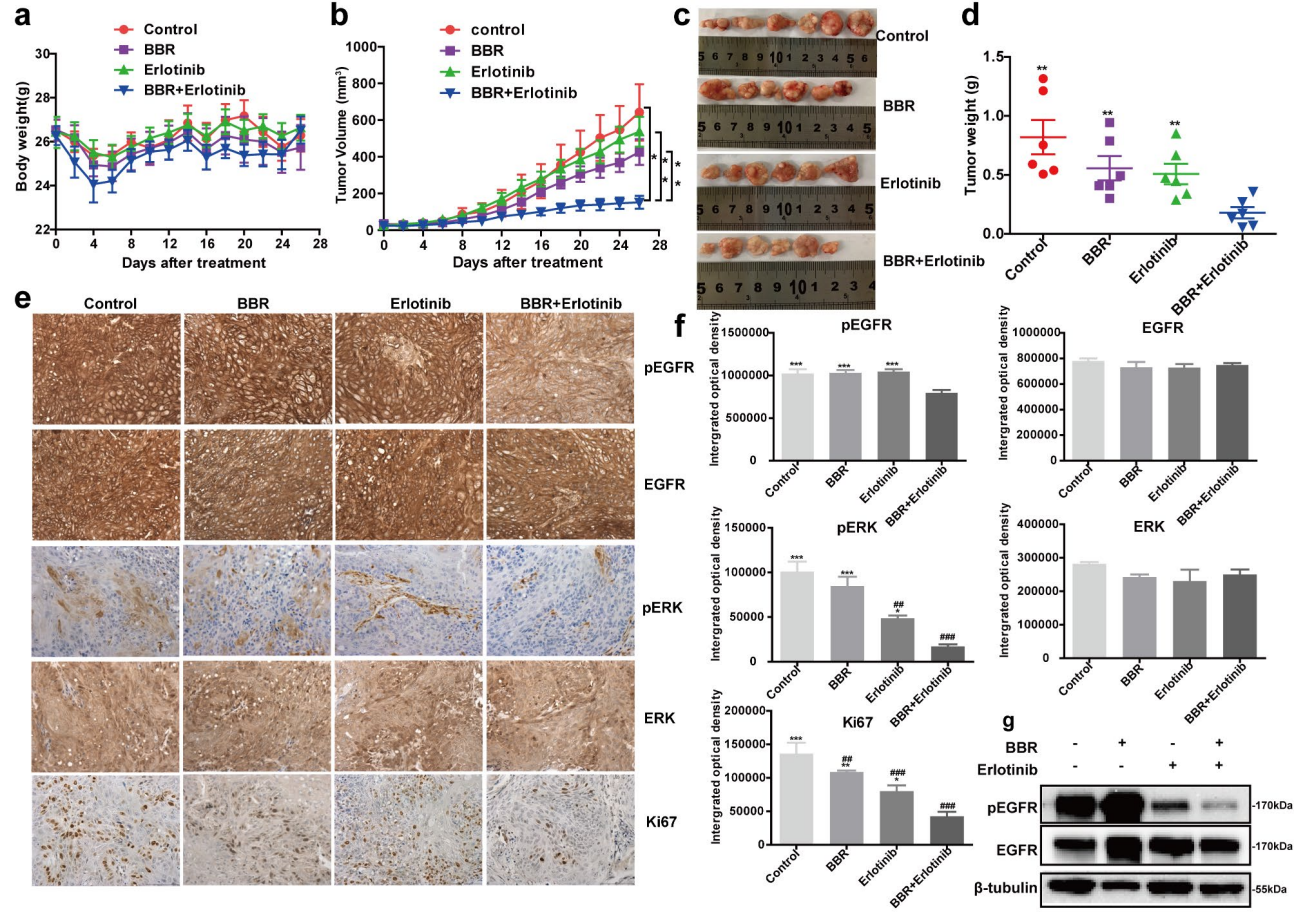
Berberine combined with erlotinib inhibited tumor growth *in vivo*. Body weights (**a)** and tumor volumes (**b)** of A431 xenograft tumors treated with vehicle control, BBR (15 mg/kg), erlotinib (25 mg/kg), and BBR plus erlotinib. (**c**) Representative photographs of tumor tissues. (**d**) Tumor weights. (**e**) Representative image of IHC (original magnification ×400). (**f**) Immunohistochemical staining analysis of pEGFR, pERK1/2, and Ki67 in A431 xenograft tumors. (**g**) The expression levels of EGFR and pEGFR in A431 xenograft tumors. Data were analyzed using one-way ANOVA and Student’s t-test, ^*^P < 0.05; ^**^P < 0.01; ^***^P < 0.001 vs. combination-therapy group. ^#^*P* < 0.05; ^##^*P* < 0.01; ^###^*P* < 0.001 vs. control

We determined the effects of BBR, erlotinib, and the combination of BBR and erlotinib on tumor growth using a xenograft model generated by subcutaneous dorsal implantations of A431 cells into nude mice. To evaluate the *in vivo* toxicity of BBR combined with erlotinib, we monitored mouse body weight. The results showed that treatment with BBR and/or erlotinib was well-tolerated without significant weight loss (*p* > 0.05, Fig. 4a). The combination of BBR plus erlotinib delayed *in vivo* tumor growth compared with that observed in the vehicle control group, as evidenced by inhibition of tumor growth in A431 cells (tumor growth inhibition (TGI) value of 78.06%). This TGI value was higher than that observed in the BBR (67.73%) and erlotinib (64.58%) groups (Fig. 4b, c and d). We evaluated the mechanisms of action of BBR and erlotinib using IHC staining. Immunohistochemistry showed that combination therapy resulted in greater inhibition of pEGFR and pERK1/2 than that in the other groups, but did not induce degradation of EGFR (*p* < 0.05). Evaluation of the expression of the proliferation marker protein Ki-67 showed that combined treatment resulted in greater inhibition of proliferation in the xenograft tumors than that observed in response to treatment with BBR or erlotinib alone (*p* < 0.05, Fig. 4e and f). Protein extracted from the xenograft tumor tissues was examined to evaluate whether the inhibition of pEGFR by treatment with BBR and erlotinib observed *in vitro* was also observed *in vivo*. Phosphorylation of EGFR was significantly inhibited by combined treatment with BBR and erlotinib in tumor samples obtained from mice (Fig. 4g).

## Discussion

Lung cancer can be categorized as non-small cell lung cancer (NSCLC) or small cell lung cancer (SCLC). Abnormal activation of EGFR is observed in approximately 50% of patients with NSCLC. Therefore, targeting EGFR has been identified as an effective anticancer strategy, and EGFR has become a well-established target for treatment of NSCLC [2, 3]. The two classes of anti-EGFR agents currently used are MAbs and TKIs. Although MAbs and TKIs exert anti-tumor effects, most patients develop drug resistance within 9–14 months [9–11]. Only a small fraction of EGFR positive advanced colorectal cancers expressing wild type KRAS respond to anti-EGFR mAbs, and acquired resistance also commonly occurs [30]. Therefore, the majority of EGFR positive cancers do not respond to TKIs or to mAb. As such, there is an obvious need to identify and develop novel treatment strategies that can complement current EGFR targeted therapy.

Berberine is an isoquinoline quaternary alkaloid derived from *Coptis chinensis* that has been used as a therapeutic agent for treatment of cancer, bacterial infections, diabetes, and cardiovascular and inflammatory diseases [31]. The EGFR signaling pathways play a critical role in proliferation, invasion, and survival. The PI3K/Akt and RAS/RAF/MEK/ERK pathways are the two main downstream pathways of EGFR signaling. Previous studies have shown that BBR decreased the phosphorylation of PI3K/AKT, ERK, and GSK3β in B16F10 melanoma cells and inhibited the activation of EGFR in these tumors [32, 33]. The specific antitumor activity of BBR on EGFR signaling pathways suggested its potential as an inexpensive, relatively non-toxic therapy when combined with EGFR-TKIs based on the premise that combination treatment could exert synergistic antitumor effects compared with monotherapies. We showed that BBR and erlotinib synergistically suppressed tumor growth by acting on the common EGFR/AKT signaling pathways in EGFR positive cells.

We used A431 cells to identify novel combination therapies targeting therapy-refractory lung cancer. We showed that combined treatment with BBR and erlotinib synergistically inhibited colony formation (Fig. 1) *in vitro* and reduced tumor growth *in vivo* (Fig. 4). Treatment with BBR and erlotinib resulted in inhibition of phosphorylation of EGFR and its downstream effectors (pEGFR-Y1045, pEGFR-Y1068, pERK, and pAKT) (Fig. 2). Berberine exhibited synergistic antitumor activity when combined with erlotinib and did not induce significant side effects in our animal experiment. Neither BBR nor erlotinib alone exerted significant therapeutic effects in the A431 model. In contrast, combination therapy led to tumor regression, significant reductions in pEGFR and pERK1/2, and significantly decreased Ki67 staining (Fig. 4). The present study suggested a novel mechanism by which the combination of erlotinib and BBR resulted in decreased EGFR pathway activity.

Members of the Bcl-2 family such as Bax and Bcl-2 play important roles in regulating the mitochondria-dependent apoptotic pathway. The Bax/Bcl-2 expression ratio is critical for induction of apoptosis [34]. Treatment of A431 cells with BBR has been shown to increase the expression of Bax and decrease the expression of Bcl-2, resulting in an increase in the Bax/Bcl-2 ratio [35, 36]. Our findings showed that treatment of A431 cells with BBR and erlotinib led to a decrease in the Bcl-2/Bax ratio (Fig. 3e and f). Epidermal growth factor receptor and its downstream effectors ERK/MAPK, AKT, STAT, and cyclin D are involved in cell proliferation and differentiation. Cyclin D may be an essential element in the pathway that connects EGFR-mediated mitogenic signals to the cell cycle at the G1/S boundary [37, 38]. Studies have shown that BBR suppressed cancer cells by inducing G1-phase cell cycle arrest and reducing cyclin D expression in lung and hepatoma cells [36, 39]. Consistent with these findings, our results showed that combined treatment inhibited the expression of phosphorylated ERK and AKT, and the expression of cyclin D in A431 cells. (Fig. 3e and f).

## Conclusion

In conclusion, our results showed that combined treatment with BBR and erlotinib synergistically inhibited A431 tumor growth *in vitro* and *in vivo*. Mechanistic studies showed that synergistic cell growth inhibition by the combination of BBR and erlotinib was associated with significantly greater inhibition of pEGFR. Combined treatment resulted in significantly greater inhibition of tumor growth through increased apoptosis and decreased cell proliferation compared to those observed in response to BBR or erlotinib alone. Our study suggested that combination therapy may provide a novel potential therapeutic strategy for treatment of EGFR positive cells.

## Supplementary Information

Not applicable.

## Data Availability

All data generated or analyzed during this study are included in this published article.

## Abbreviations

Bax: Bcl-2-associated X protein
Bcl-2: B cell lymphoma 2
DMEM: Dulbecco’s Modified Eagle’s Medium
DMSO: dimethyl sulfoxide
MTT: 3-(4,5-dimethylthiazol-2-yl)-2,5-diphenyltetrazolium
EGFR-TKIs: epidermal growth factor receptor tyrosine kinase inhibitors
BBR: Berberine
